# Problematic Alcohol Use Among Adolescents in Germany: Representative Cross-Sectional Study

**DOI:** 10.2196/87800

**Published:** 2026-02-06

**Authors:** Rebekka Schröder, Tim Hamer, Ralf Suhr, Lars König

**Affiliations:** 1 Stiftung Gesundheitswissen Berlin Germany

**Keywords:** adolescents, alcohol, Germany, health literacy, problematic alcohol use, representative survey

## Abstract

**Background:**

Alcohol is a widely used psychoactive substance, and its use constitutes a major public health challenge due to its immediate and long-term adverse effects on various health-related outcomes. Adolescence has been identified as a particularly vulnerable phase regarding alcohol use. Although consumption rates in this age group have declined in Germany over the past decades, a plateau has been reached, and there is a continued need for interventions to further reduce consumption rates.

**Objective:**

This study aimed to assess problematic alcohol use among adolescents in Germany and explore associations with sociodemographic and psychosocial characteristics, particularly with health literacy, to inform future interventions tailored to the specific needs of this target group.

**Methods:**

In a cross-sectional quota-based survey, 2006 adolescents (aged 12-17 years) completed an online survey (n=1406) or face-to-face interview (n=600) assessing the frequency of weekly alcohol use, the presence of problematic alcohol use (German version of the Car-, Relax-, Alone-, Forget-, Friends-, Trouble- questionnaire [CRAFFT-d]), sociodemographic information, and health literacy (European Health Literacy Survey instrument [HLS-EU-Q16]). Based on their CRAFFT-d and HLS-EU-Q16 scores, participants were identified as exhibiting problematic alcohol use (vs no problematic alcohol use) and inadequate or problematic health literacy levels (vs adequate health literacy levels), respectively. Chi-square tests were computed to analyze differences between different groups (as defined by the sociodemographic factors, weekly alcohol consumption frequency, and health literacy) in terms of problematic alcohol use (binary CRAFFT-d outcome).

**Results:**

Approximately 20% (390/2006) of the participants reported consuming alcohol on at least 1 day per week, and 12.7% (255/2006) of the sample met the CRAFFT-d screening criterion for problematic alcohol use. Problematic alcohol use was significantly associated with gender (*χ*^2^_1_=20.96, *V*=0.10; *P*<.001), age (*χ*^2^_2_=85.88, *V*=0.21; *P*<.001), subjective social status (*χ*^2^_2_=8.23, *V*=0.06; *P*=.02), and migration background (*χ*^2^_1_=5.60, *V*=0.05; *P*=.02), but there were no significant associations with level of education (*χ*^2^_1_=3.43, *V*=0.04; *P*=.06), and health literacy (*χ*^2^_1_=1.54, *V*=0.03; *P*=.21). In addition, participants who reported more frequent alcohol consumption per week, also met the screening criterion for problematic alcohol consumption more frequently (*χ*^2^_7_=698.65, *V*=0.59; *P*<.001).

**Conclusions:**

The findings demonstrate that problematic alcohol use is more common in boys than girls, in older vs younger adolescents, in those with high or low (vs intermediate) social status, in individuals with (vs without) a migration background, and in those who drink alcohol more frequently. These results emphasize the necessity of implementing targeted prevention strategies that address the specific risk profiles of adolescents concerning alcohol consumption.

## Introduction

### Background

Alcohol use poses a major public health challenge [1,2], and adolescence has emerged as a particularly vulnerable period for its detrimental short- and long-term effects [3-5]. This study addresses the problematic use of alcohol among adolescents in Germany and how this problematic use relates to psychosocial and sociodemographic factors with cross-sectional data.

### Public Health Relevance of Alcohol Use

Alcohol is one of the most widely used psychoactive substances worldwide [6,7]. At the same time, more than 3 million deaths worldwide are attributable to alcohol, corresponding to 5.3% of all deaths [2]. This effect is exacerbated in younger age, with 18.4% of all deaths in men aged 25-29 years attributed to alcohol. Women are less affected than men but also experience a substantial health burden due to alcohol [2]. While there is some evidence that alcohol might have beneficial effects when consumed in smaller quantities [8-10], this position has been challenged in recent years and might be attributed to methodological artifacts [11-13]. Importantly, it is undisputed that health effects are detrimental when alcohol is consumed in large quantities [10,14-16]. These harmful effects on health can be observed in the short and long term [11]. Short-term effects refer to the immediate consequences of alcohol consumption, including acute alcohol intoxication as well as decreased visuomotor coordination, increased reaction times, and increased impulsivity [11,17,18]. These may lead to more severe consequences, such as injuries or deaths, for example, from falls, traffic accidents, or drowning [11,19]. The arguably most apparent long-term effects of excessive alcohol consumption are clinically relevant alcohol abuse and dependence, which are highly prevalent worldwide, and also in many European countries, including Germany [1,20]. These effects are associated with a significant decline in physical and mental health and a loss of quality of life [21,22]. Other long-term effects of alcohol consumption on health-related outcomes include, but are not limited to, cancer, cardiovascular, gastrointestinal, and neuropsychiatric conditions, which may result in premature mortality [11,16]. In addition to these individual consequences, alcohol consumption has also been associated with substantial direct and indirect economic costs at a societal level [23].

### Alcohol Use Among Adolescents

Given the detrimental effects of alcohol consumption on various health outcomes, more recent nutritional policies in Germany recommend abstaining from alcohol entirely [11]. However, alcohol consumption remains at a high level in Germany compared to other Western-European countries [24,25]. Critically, alcohol is consumed from a young age [26]. Recent evidence from representative samples estimates the prevalence of regular alcohol use, defined as consumption of alcoholic beverages at least weekly in the past 12 months, at about 9.7% in those aged 12-17 years, with substantially higher rates in boys (12.4%) than girls (6.9%). In addition, 63% of all adolescents in that age group report lifetime alcohol use, and about 4% show risky drinking behaviors, exceeding established thresholds for risky alcohol consumption in adults (equivalent to drinking more than 12 grams of pure alcohol for women and 24 grams of pure alcohol for men daily). However, a decrease in general and risky alcohol consumption over the past decades has also been observed in adolescents [26]. Crucially, this decline appears to have plateaued in the last few years, showing only minimal variation [26].

### Problematic Alcohol Use Among Adolescents and Its Correlates

The stagnation in alcohol use in recent years underscores the need for renewed public health strategies to address the persistent harm associated with alcohol consumption from a young age. A particular focus should be placed on problematic alcohol use in adolescents as this has been associated with severe consequences, including functional and structural brain damage and increased risks for developing alcohol use disorders [3-5,27]. A recent investigation with data from a representative sample from Germany found that 11.3% of the participants aged 12-17 years met the criteria for problematic alcohol use on a standardized screening tool. Moreover, it was shown that problematic alcohol use was associated with greater psychopathology, lower mindfulness, and lower quality of life, even when potentially confounding factors were controlled for [28]. In order to develop interventions that are specifically tailored to societal needs, particularly for adolescents, it is necessary to collect more detailed data on the associations between problematic alcohol use and other factors.

In particular, there is currently insufficient data on the relationship between problematic alcohol consumption and specific sociodemographic and psychosocial factors. For example, the association with health literacy has not yet been adequately explored. Health literacy encompasses the knowledge, motivation, and competencies in the process of accessing, understanding, appraising, and applying health information [29]. Health-literate individuals are more likely to successfully take care of their own health and navigate the health care system [29]. As a consequence, individuals with greater health literacy often exhibit healthier lifestyles (eg, adequate medication intake, less sedentary behavior), have a better overall health status, and lower mortality than individuals with lower health literacy levels [29-32]. This might also give them an advantage when making decisions regarding their alcohol consumption [33,34]. For example, greater levels of health literacy may help individuals to understand and appraise content, units, strengths, and harms of alcohol and make healthier choices regarding their alcohol intake [35,36].

Furthermore, while some relevant sociodemographic correlates (eg, gender) of (problematic) alcohol use among adolescents have already been obtained [26], there is still insufficient data on other possible associations and a strong need for replication of prior findings. Particularly, some associations have not been found consistently, for example, concerning levels of education and social status [37-40].

### Study Aims

Therefore, the aim of this study was to assess the frequency of alcohol use and the prevalence of individuals with problematic alcohol use among adolescents in Germany, and to explore associations between problematic alcohol use and various sociodemographic and psychosocial characteristics, including health literacy.

## Methods

### Participants, Recruitment, and Survey Methodology

The target population for this study was adolescents aged 12-17 years living in Germany with sufficient knowledge of the German language to participate in the study. We aimed to recruit 2000 individuals in order to allow precise estimates of prevalences of problematic drinking, to test for differences between the sociodemographic and psychosocial groups, and to allow representativeness of the sample for the characteristics described below (more information is given in the Data Weighting section). For example, an a priori power analysis for a chi-square test of independence for comparing the prevalences of problematic drinking across different age groups yielded a minimum sample size of 1283 participants (input parameters: 80% power, α=.05, *df*=5, effect size ω=0.1 [41]). Recruitment of the participants, study administration, and data acquisition were carried out by the German market research institute Gesellschaft für Innovative Marktforschung mbH and took place from November 2024 to January 2025. Only complete datasets without missing data were provided by the market research institute. A total of 2006 individuals participated in the study. Data were collected both online via web-based surveys (n=1406 participants, 70.1% of all participants) and via in-person face-to-face interviews (n=600 participants, 29.9% of all participants). Online participants were recruited from 3 online-access panels via personalized e-mail invitation links. Face-to-face participants were recruited from validated and regularly updated address pools of private households in Germany with known household composition. All participants aged 15 and younger were recruited via their parents. A quota-based sampling strategy was used, with quotas for 3 age categories (12-13 years, 14-15 years, and 16-17 years), gender, type of school, and federal state. Quotas were based on current data from the MA Audio (agma) study and the Federal Statistical Office (Statistisches Bundesamt). The MA Audio study is a large nation-wide and representative study with more than 66,000 yearly interviews. Detailed descriptive statistics for the final sample are in Table 1.

### Data Weighting

Despite careful quota-based recruitment, some disparities between the sample and the reference population regarding relevant sociodemographic characteristics remain, for example, because some sociodemographic characteristics, such as level of education, negatively correlate with the willingness to participate in online surveys. Therefore, the data were weighted by the market research institute to match the target distribution of relevant reference studies (again using the latest MA Audio study and specifications of the Federal Statistical Office). The reference population for the weighting procedure is the German-speaking population living in Germany aged 12 years and older. The following weighting variables and combinations were used: age × gender; education (operationalized as type of school); and federal state. The calculation method involved using an iterative process in which all weighting variables of the sample are used simultaneously to inform the weighting with the aim of achieving minimal weights. This procedure resulted in a single weighting factor per individual.

### Measures

#### Sociodemographic Information

We collected sociodemographic information concerning gender (boys, girls, and diverse), age, level of education, subjective social status, and migration background. For further analyses, participants were categorized into 3 age groups (12-13 years, 14-15 years, and 16-17 years).

To assess levels of education, participants were asked to indicate the type of school they currently attend. They were grouped into 2 categories: those participants attending the most advanced type of secondary school (Gymnasium, equivalent to grammar schools where a university entrance qualification can be obtained) were assigned a high level of education, and all other participants were assigned a low level of education.

Subjective social status was assessed using the German version of the MacArthur scale [42,43]. The scale uses the analogy of a ladder to represent social status, with the top rung (10) symbolizing the highest social status and the bottom rung (1) denoting the lowest social status. Participants are asked to identify the rung that best represents their individual position relative to other members of society. In total, 3 categories of subjective social status were determined: low subjective social status (scores 1-4), intermediate subjective social status (scores 5-7), and high subjective social status (scores 8-10).

Finally, participants were asked to specify whether they had a migration background, which was defined as having at least 1 parent who was not born in Germany or having been born outside of Germany themselves.

#### Health Literacy

The German translation of the short version of the European Health Literacy Survey instrument (HLS-EU-Q16) was administered to assess health literacy [44-46]. Participants were asked to indicate perceived difficulty in accessing, understanding, appraising, and applying information in the domains of health care, disease prevention, and health promotion. A total of 16 items was presented with a 4-point Likert scale (“very easy,” “fairly easy,” “fairly difficult,” and “very difficult”). Individual data points were preprocessed by dichotomizing item responses, that is, by assigning one point to any “fairly easy” and “very easy” responses and zero points to any “fairly difficult” and “very difficult” responses. Then, a total score was calculated by summing up points across these dichotomized items (possible range 0-16). Finally, participants were grouped according to their overall score, for example, those with inadequate or problematic health literacy (scores 0-12) and those with adequate health literacy (scores 13-16) [47]. The HLS-EU-Q16 questionnaire was originally developed from a more comprehensive questionnaire with 47 items. It has been thoroughly validated and analyzed regarding its reliability in samples of the (adult) general population [45,46]. In addition, the 16-item version of the questionnaire was found to be both valid and reliable in studies of adolescents [48,49].

#### Frequency of Alcohol Use and Problematic Alcohol Use

Two relevant statistics were collected regarding the alcohol use of the participants. First, to assess the frequency of alcohol consumption, participants were asked to indicate in a closed-response format with 8 response options (0-7 days) on how many days they consume alcohol in an average week. Second, to assess problematic alcohol use, the German version of the Car-, Relax-, Alone-, Forget-, Friends-, Trouble- (CRAFFT-d) questionnaire [50,51] was administered. The questionnaire’s name is an acronym for its 6 items used to assess problematic alcohol use. The original questionnaire was developed as a screening instrument for both alcohol and drug misuse in adolescents [51], but the German CRAFFT-d only assesses alcohol use [50]. In 6 items the participants are asked to indicate whether they have ever ridden in a car with a driver (including themselves) who had consumed alcohol, whether they ever drink to relax, feel better about themselves or fit in, whether they have ever drunk by themselves, whether they have ever forgotten things while using alcohol, whether they were ever told by friends or family to cut down drinking, and whether they had ever gotten into trouble while drinking [51]. Each item is a simple yes-no question, and an overall score can be calculated as the number of positive answers to these questions. The outcome of the questionnaire is binary: if the overall score of an individual is equivalent to or exceeds 2, these individuals are classified as having potentially problematic alcohol consumption; a score lower than 2 indicates no problematic alcohol consumption [50-53]. The German CRAFFT-d has been validated with a sensitivity of 88.8% and a specificity of 66.2% [53].

### Statistical Analysis

Data preprocessing and all statistical analyses were conducted with the statistical software SPSS (version 29.0.2.0; IBM Corp). All inferential statistical analyses are reported for the weighted data (Data Weighting for details on the weighting procedures). Internal consistencies of the CRAFFT-d and HLS-EU-Q16 were calculated as Cronbach α. To test for significant associations between the sociodemographic and psychosocial factors and problematic alcohol consumption according to the CRAFFT-d screening criterion, chi-square tests of independence were calculated with Cramer *V* as a measure of effect size. Cramer *V* assesses the strength of the association between 2 nominal variables, for example, when analyzing contingency tables. It can be interpreted according to the following rule of thumb: 0.10 small, 0.30 medium, and 0.50 large [54].

### Ethical Considerations

The ethics committee of the Berlin Medical Association did not raise any ethical or professional objections to the study protocol (reference number Eth-SB-24-047). Informed consent was obtained from all participants before data collection was initiated. For all participants aged 15 and younger, informed consent was also provided by a parent or legal guardian. The market research institute provided only anonymized data to the Stiftung Gesundheitswissen. Confidentiality was maintained throughout the study. Participants were not directly compensated by the Stiftung Gesundheitswissen. They received panel-specific compensation or credits that can be redeemed for bank transfers, vouchers, or raffle entries. In face-to-face settings, compensation is handled individually by the interviewers and typically involves direct cash payments or small gifts. This study was part of a larger study that assessed several different health-related constructs and behaviors (eg, anxiety and eating habits) with a specific focus on health literacy in 2 independent samples of the adult general population and adolescents in a cross-sectional study design. Further analyses focusing on other thematic areas are ongoing and are expected to result in additional publications.

## Results

### Sample Characteristics and Descriptive Statistics

A total of 2006 individuals aged 12-17 years (mean 14.47, SD 1.70 years) participated in the study. Detailed descriptive statistics of the sample across the relevant sociodemographic and psychosocial categories before and after the weighting process are presented in Table 1.

**Table 1:** Sample characteristics of weighted and unweighted data from a cross-sectional survey (2024-2025) of N=2006 adolescents (aged 12-17 years) in Germany.

Variables^a^			Unweighted sample, n (%)		Weighted sample, n (%)
**Gender**					
	Boys	1025 (51.1)		1035 (51.6)	
	Girls	975 (48.6)		964 (48.1)	
	Diverse	6 (0.3)		6 (0.3)	
**Age groups (years)**					
	12-13	657 (32.8)		676 (33.7)	
	14-15	688 (34.3)		681 (34)	
	16-17	661 (33)		649 (32.4)	
**Level of education**					
	Low	1167 (58.2)		1370 (68.3)	
	High	839 (41.8)		636 (31.7)	
**Social status**					
	Low	248 (12.4)		258 (12.9)	
	Intermediate	1324 (66)		1329 (66.3)	
	High	434 (21.6)		419 (20.9)	
**Migration background**					
	Yes	292 (14.6)		295 (14.7)	
	No	1714 (85.4)		1711 (85.3)	
**Health literacy**					
	Inadequate or problematic	1180 (58.8)		1203 (60)	
	Adequate	826 (41.2)		803 (40)	

^a^Cumulative percentages and absolute numbers may exceed or fall below 100% due to weighting and rounding.

### Reliability of the Measures

Internal consistency of the CRAFFT-d overall score to assess problematic alcohol use was α=.75, and internal consistency of the HLS-EU-Q16 to assess health literacy was α=.86.

### Descriptive Findings on Frequency of Alcohol Consumption

On average, participants drank alcohol on a mean of 0.35 (SD 0.89) days per week, with the majority of the participants (1615/2006, 80.5%) reporting not drinking at all across an average week. Details of the frequency of alcohol consumption are presented in Table 2.

**Table 2:** Frequency of weekly alcohol consumption across the sample of 2006 adolescents (aged 12-17 years) from a cross-sectional survey (2024-2025) in Germany.

How many days per week, on average, do you consume alcohol?^a^	Values, n (%)
0	1615 (80.5)
1	221 (11)
2	91 (4.6)
3	46 (2.3)
4	15 (0.8)
5	7 (0.4)
6	6 (0.3)
7	4 (0.2)

^a^Note: Cumulative percentages and absolute numbers may exceed or fall below 100% due to weighting and rounding.

Across the entire sample, n=255 (12.7%; 95% CI 11.2%-14.2%) individuals reported problematic alcohol consumption according to the CRAFFT-d screening questionnaire, and n=1751 (87.3%) individuals did not. Response frequencies for each CRAFFT-d item are reported in Table 3. The items referring to drinking to relax, forgetting things when drinking, and getting into trouble while drinking were affirmed more frequently (ie, by more than 10% of the sample) than the items on drunk driving, drinking alone, and being told to cut down drinking, which were affirmed by less than 10% of the sample. On average, participants reached a sum score of mean 0.50 (SD 1.11) in the CRAFFT-d questionnaire.

**Table 3:** Individual item responses for the German version of the Car-, Relax-, Alone-, Forget-, Friends-, Trouble- questionnaire to assess problematic alcohol use across the sample of N=2006 adolescents (aged 12-17 years) from a cross-sectional survey (2024-2025) in Germany.

Item number	Items^a^	Yes, n (%)	No, n (%)
1	Have you ever ridden in a car driven by someone (including yourself) who had been using alcohol?	113 (5.6)	1893 (94.4)
2	Do you ever use alcohol to relax, feel better about yourself, or fit in?	209 (10.4)	1797 (89.6)
3	Do you ever use alcohol when you are by yourself (alone)?	104 (5.2)	1902 (94.8)
4	Do you ever forget things you did while using alcohol?	209 (10.4)	1797 (89.6)
5	Do your family or friends ever tell you that you should cut down on your drinking?	166 (8.3)	1840 (91.7)
6	Have you ever gotten into trouble while you were using alcohol?	205 (10.2)	1801 (89.8)

^a^In contrast to the original English version, the German CRAFFT-d only assesses alcohol consumption and does not refer to drug consumption [50,51]. Therefore, the items are reported with adjusted wording to match the German version, which was presented to the participants in this study. Cumulative percentages and absolute numbers may exceed or fall below 100% due to weighting and rounding.

### Prevalences of Problematic Alcohol Use According to the CRAFFT-d Questionnaire and Associations With Sociodemographic and Psychosocial Variables

Table 4 presents the descriptive results regarding rates of problematic and nonproblematic alcohol use according to the CRAFFT-d questionnaire across the sociodemographic and psychosocial categories.

Boys reported significantly more problematic alcohol consumption than girls (*χ*^2^_1_=20.96, *V*=0.10; *P*<.001). Participants with diverse genders were not included in this analysis because cell frequencies were too low to allow testing.

Problematic alcohol consumption significantly increased with increasing age (*χ*^2^_2_=85.88, *V*=0.21; *P*<.001). Participants aged 12-13 years had the lowest frequency of problematic alcohol consumption (34/676, 5%), and those aged 16-17 years had the highest frequency of problematic alcohol consumption (142/649, 21.9%), with participants aged 14-15 years falling in between these 2 groups (79/681, 11.6%).

Problematic alcohol consumption was not significantly associated with level of education (χ^2^_1_=3.43, *V*=0.04; *P=*.06), with a descriptively marginally higher frequency in those with a low (187/1370, 13.6%) compared to a higher level of education (68/636, 10.7%).

Participants who reported low subjective social status had the highest frequency of problematic alcohol consumption (46/258, 17.8%), followed by participants with high subjective social status (56/418, 13.4%) and intermediate subjective social status (152/1329, 11.4%). This association was significant (*χ*^2^_2_=8.23, *V*=0.06; *P*=.02).

Problematic alcohol consumption was also significantly associated with migration background, with a higher rate of problematic alcohol consumption in individuals with a migration background (50/295, 16.9%) compared to those without a migration background (205/1711, 12%; *χ*^2^_1_=5.60, *V*=0.05; *P*=.02).

Problematic alcohol consumption was not significantly associated with levels of health literacy (*χ*^2^_1_=1.54, *V*=0.03; *P*=.21). There was a descriptively higher rate of problematic alcohol consumption in individuals with inadequate or problematic health literacy (162/1203, 13.5%) than in those with adequate health literacy (93/803, 11.6%).

Participants who reported more frequent alcohol consumption per week also showed more frequent problematic alcohol consumption (*χ*^2^_7_=698.65, *V*=0.59; *P*<.001). Rates of problematic alcohol consumption were lowest in those who reported drinking on average on zero days per week (57/1614, 3.5%), and highest in those who drank every day (4/4, 100%), with those drinking on 2 to 6 days per week falling in between these 2 extremes (Table 4). However, in some cells, total frequencies were very low, which should be kept in mind when interpreting the results.

**Figure 1:**
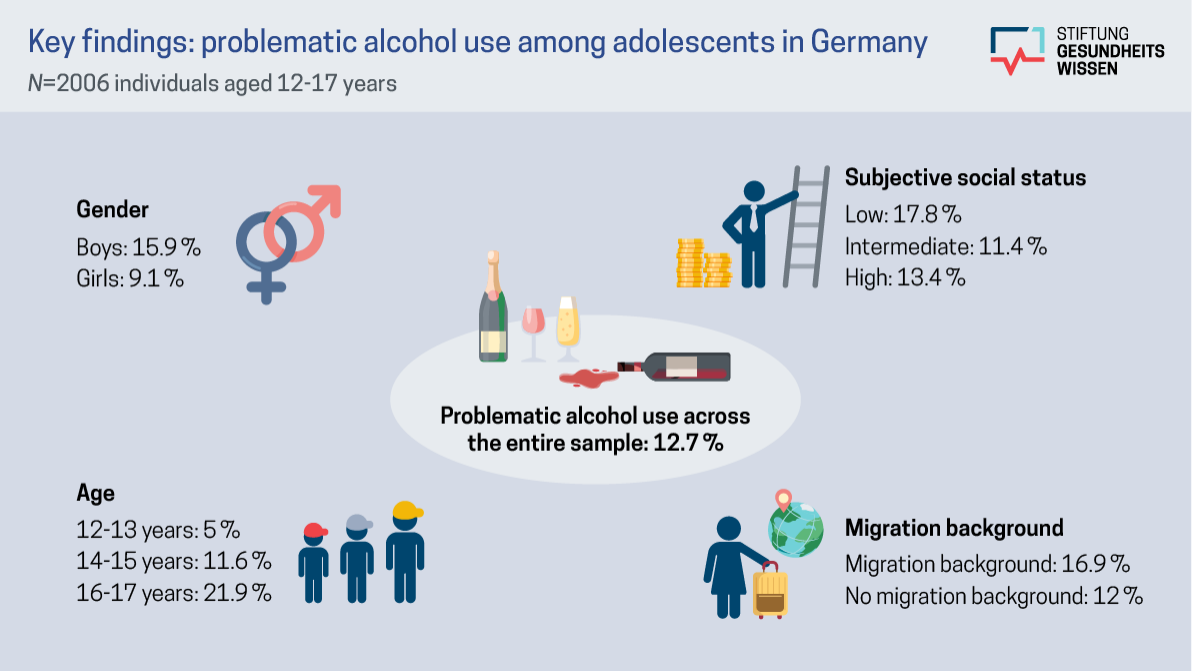
Visual summary of the key findings regarding problematic alcohol use in a sample of N=2006 adolescents (aged 12-17 years) from a cross-sectional survey (2024-2025) in Germany.

A summary of the key significant findings is presented in Figure 1.

**Table 4:** Proportions of positive and negative screens obtained with the German version of the Car-, Relax-, Alone-, Forget-, Friends-, Trouble-questionnaire indicating problematic alcohol use across the sociodemographic and psychosocial categories in a sample of N=2006 adolescents (aged 12-17 years) from a cross-sectional survey (2024-2025) in Germany.

Categories^a^		Problematic alcohol use, n (%)	No problematic alcohol use, n (%)
Overall		255 (12.7)	1751 (87.3)
**Gender**			
	Boys (n=1035)	165 (15.9)	870 (84.1)
	Girls (n=964)	88 (9.1)	876 (90.9)
**Age groups (years)**			
	12-13 (n=676)	34 (5)	642 (95)
	14-15 (n=681)	79 (11.6)	602 (88.4)
	16-17 (n=649)	142 (21.9)	507 (78.1)
**Level of education**			
	Low (n=1370)	187 (13.6)	1183 (86.4)
	High (n=636)	68 (10.7)	568 (89.3)
**Social status**			
	Low (n=258)	46 (17.8)	212 (82.2)
	Intermediate (n=1329)	152 (11.4)	1177 (88.6)
	High (n=418)	56 (13.4)	362 (86.6)
**Migration background**			
	Yes (n=295)	50 (16.9)	245 (83.1)
	No (n=1711)	205 (12)	1506 (88)
**Health literacy**			
	Inadequate or problematic (n=1203)	162 (13.5)	1041 (86.5)
	Adequate (n=803)	93 (11.6)	710 (88.4)
**Frequency of weekly alcohol consumption**			
	Zero days (n=1614)	57 (3.5)	1557 (96.5)
	One day (n=221)	88 (39.8)	133 (60.2)
	Two days (n=91)	48 (52.7)	43 (47.3)
	Three days (n=46)	34 (73.9)	12 (26.1)
	Four days (n=15)	13 (86.7)	2 (13.3)
	Five days (n=7)	5 (71.4)	2 (28.6)
	Six days (n=6)	4 (66.7)	2 (33.3)
	Seven days (n=4)	4 (100)	0 (0)

^a^Cumulative percentages and absolute numbers may exceed or fall below 100% due to weighting and rounding.

## Discussion

### Principal Findings

The aim of this study was to assess current data on the rates of problematic alcohol use and the frequency of alcohol use among adolescents aged 12-17 years in Germany, and to explore how problematic alcohol use relates to sociodemographic and psychosocial factors as well as frequency of alcohol use. Approximately 20% (390/2006) of all participants reported drinking alcohol on at least 1 day per week, and 12.7% (255/2006) of the sample met the CRAFFT-d screening criterion for problematic alcohol use. Problematic alcohol use was significantly associated with gender, age, subjective social status, migration background, and frequency of weekly alcohol consumption. However, there were no significant associations between problematic alcohol consumption and level of education or health literacy.

The fact that about 20% (390/2006) of the sample reported alcohol consumption on at least 1 day in an average week is concerning, as dose-response relationships between levels of alcohol use and alcohol use disorder incidence and mortality have been found in previous studies [55]. Consistent with this line of evidence, the frequency of alcohol consumption was associated with problematic alcohol use in our data, with higher rates of problematic use observed in those individuals who consumed alcohol more frequently across an average week. As a recent meta-analysis using longitudinal data suggests that even small doses of alcohol are associated with an increased risk of developing an alcohol use disorder and increased mortality [55], there is a clear public health interest in further reducing alcohol consumption. Regarding the prevalence of problematic alcohol use, our findings obtained with the CRAFFT-d questionnaire (255/2006, 12.7%) are comparable to recent findings that found a rate of 13.6% (using a liberal threshold) or 11.3% (using a more conservative threshold) of problematic alcohol use with a different screening instrument in a sample of more than 4000 adolescents from Germany [28], highlighting the validity of our approach. Moreover, when compared to data from 2015 from slightly older samples (CRAFFT-d baseline means of 0.95, SD 1.19 and 1.10, SD 1.29 in 2 groups), we obtained lower CRAFFT-d mean scores (mean 0.50, SD 1.11). This pattern of results converges with our finding of higher scores in older individuals and with a decline in the frequency of adolescent drinking in recent years [26]. Overall, our findings demonstrate that there is still a substantial proportion of adolescents (255/2006, 12.7%) who show problematic drinking patterns. Examining the CRAFFT-d items individually reveals that approximately 1 in 10 adolescents have experienced trouble because of drinking, have forgotten things due to alcohol use, and have drunk to relax, feel better, or fit in. The other items concerning drunk driving, having been told to cut down on drinking, and drinking alone were affirmed less frequently, but still had prevalence rates exceeding 5% in the sample. These results emphasize the need for continued and intensified evidence-based efforts to reduce alcohol consumption and problematic drinking among adolescents in Germany.

When analyzing the reasons why adolescents drink alcohol, 3 major motives emerge in the literature: social enhancement (eg, to obtain social rewards), coping (eg, to attenuate negative affect), and dominance (eg, to obtain respect) [56]. In addition, on a neurobiological level, adolescents might be particularly sensitive to the rewarding effects of alcohol and less sensitive to its sedative effects [57]. Crucially, adolescence is characterized as a period of rapid changes in various areas of life, including physical, social, hormonal, and mental transitions, during which individuals have to cope with multiple developmental tasks [58,59]. These transitions also manifest in concrete behaviors, such as tendencies to take more risks, which might be explained by immature self-regulation competencies and changes in personality factors such as sensation seeking and impulsivity. Of note, these factors have been found to influence alcohol use in longitudinal studies [60-62]. Moreover, alcohol expectancies, that is, the individual beliefs about alcohol effects, have been shown to change in young adolescence and to predict alcohol use in that age group. Environmental factors (eg, influences from parents and peers) play an important role in the development of these expectancies [63], and there is first evidence that programs that include peer groups might be effective when tackling alcohol use in adolescents [64]. Taken together, the findings on predictors of alcohol use among adolescents suggest that psycho-educative and other efforts to reduce alcohol use in that age group might be more effective when they involve the social environment of the targeted population and when they particularly target alcohol expectancies and individual motives to drink [56,63,64], but more research is needed to make causal claims on these relationships.

Problematic alcohol use was associated with some—but not all—sociodemographic factors assessed in our study. For example, problematic alcohol use was found to be more prevalent among boys than girls, consistent with the finding that boys generally consume alcohol more frequently than girls [26]. This finding suggests that gender-specific prevention strategies may be a viable option that should be further investigated in the future [65].

Problematic alcohol consumption was more frequent in the older (16-17 years) compared to the intermediate (14-15 years) and the younger age groups (12-13 years). In the oldest adolescents, problematic alcohol use was observed in more than 1 in 5 participants. Although problematic drinking rates were lower in the youngest age group (5%), the reported frequency in these individuals is still concerning, as alcohol consumption is associated with detrimental developmental consequences [3,4]. Moreover, early adolescence is a particularly vulnerable period as drinking patterns in early adolescence predict problematic alcohol consumption later in life [66,67]. Therefore, prevention strategies specifically targeting both young and older adolescents should be further developed and evaluated [68].

Concerning subjective social status, frequency of problematic alcohol use followed a V-shaped pattern, with the most frequent problematic use in those with the lowest and highest subjective social status and the least frequent problematic consumption in those with intermediate subjective social status. However, problematic drinking was not associated with the level of education in our sample. This pattern of results is only partly in line with evidence that demonstrates that general alcohol consumption is typically higher in adolescents with lower compared to higher socioeconomic status [37] and that it is more often harmful (eg, leading to more frequent hospitalizations) in these individuals [38]. However, other studies point to opposite associations and higher alcohol consumption in individuals with higher socioeconomic status [39] or, in the case of binge drinking, do not find any clear association [40]. Increased problematic consumption among adolescents from higher socioeconomic levels might be explained by cultural differences, financial resources to buy alcohol, and the availability of alcohol at home [39], whereas more harmful alcohol consumption in individuals with a lower socioeconomic background might be attributed to a lack of parental support or monitoring alongside increased distress among the adolescents [38,69]. When interpreting these results, it should be kept in mind that the evidence comes from studies in different samples, from different cultural backgrounds, in different age groups, and using different modes to assess social or socioeconomic status, which makes it difficult to compare these findings. Our results add to the literature by demonstrating that among adolescents aged 12-17 years in Germany, both low and high subjective social status are associated with higher problematic alcohol use. Furthermore, the level of education was not associated with problematic alcohol use.

More frequent problematic alcohol use was observed in individuals with a migration background compared to those without a migration background. These findings can be further elucidated with the help of evidence from a recent study assessing both risky alcohol consumption (exceeding hazardous dose thresholds concerning total alcohol content) and binge drinking (exceeding thresholds for the number of drinks consumed on a single occasion) in a sample similar to ours. The study found that there are interindividual differences between individuals from various cultural backgrounds concerning their drinking patterns [26]. Risky use rates were similar in individuals with no migration background and those from Western Europe, Eastern Europe, and Turkey or Asia, but significantly lower in individuals from other regions. In contrast, binge drinking was more prevalent in individuals from Western Europe compared to those with no migration background, but less prevalent (compared to those with no migration background) in individuals from Eastern Europe and other countries, while there was no difference between those with no migration background and individuals from Turkey or Asia. Future studies need to investigate more comprehensively how problematic drinking is associated with specific cultural backgrounds and how this information can be used to develop culturally sensitive prevention strategies.

Interestingly, problematic alcohol use was not associated with general health literacy. This finding is surprising, given that health literacy predicts many other health-related behaviors [31,70] and has been shown to be associated with alcohol-related behaviors in adult populations [33,34]. It is possible that the administered instrument to measure general health literacy was too broad to capture its specific facets that more closely apply to the use of alcohol. In this context, recently, the term “alcohol health literacy,” or “alcohol literacy,” has been introduced and conceptualized. While its precise definition is still subject to ongoing debate, it appears to closely relate to general health literacy and more specifically focuses on the “capacity to obtain, process, and understand knowledge about alcohol content, units, strengths, and harms” [35,36]. First attempts to measure alcohol health literacy have been made in adults, but to date, there is no data on levels in adolescents, which should be addressed in future research [71]. Recently, new recommendations have been published to increase alcohol health literacy and reduce alcohol consumption in Germany [35]. These recommendations include action on the levels of education and information, in the health care system, and concerning alcohol control policy. One important pillar concerning education and information is the implementation of effective alcohol prevention programs in schools and the provision of easily accessible information about alcohol, especially for adolescents and young adults [35,72].

### Limitations

When interpreting the results of this study, it is important to bear in mind the following limitations. First, as noted above, we used a measure of general health literacy that might not be specific enough to measure relevant aspects of alcohol-related health literacy in the present sample [35,36]. Second, this was a cross-sectional study, which means that the observed associations cannot be interpreted as causal relationships [73]. Third, problematic alcohol use was assessed with a short screening questionnaire, which does not replace a formal clinical diagnosis for alcohol misuse or dependence. However, importantly, the questionnaire has been validated thoroughly with high sensitivity and specificity scores and therefore provides good estimates for problematic drinking [51-53,74]. Fourth, problematic alcohol use was assessed with a self-report questionnaire. As there is some level of stigma concerning alcohol use, especially among minors who are not legally allowed to drink, there is always a risk of bias due to social desirability and impression management even in anonymous surveys [75]. This might have led to an underestimation of the actual prevalence of problematic drinking in our sample [75]. Fifth, we only collected data on the frequency of drinking and on potential problematic drinking, but not on drinking quantity, which would have allowed us to conduct more in-depth analyses. Finally, the proportion of individuals with a migration background was lower than the most recent census data from Germany indicates. This discrepancy might be attributed to insufficient knowledge of the German language and the more challenging recruitment of this population segment. Therefore, our results concerning migration background should be interpreted cautiously.

### Conclusions

This study presents data on problematic alcohol consumption among adolescents in Germany in addition to examining its associations with various psychosocial and sociodemographic factors. The findings show that problematic alcohol use is more prevalent among boys than girls, among older age groups than younger ones, among individuals with a higher or lower subjective social status than those with an intermediate subjective social status, among participants with a migration background than those without, and among those who consume alcohol more frequently on a weekly basis. However, there were no significant associations between problematic alcohol use and levels of education or health literacy. These results underscore the importance of targeted prevention strategies that address the specific risk profiles of adolescents. By tailoring interventions to individuals with a higher risk, policymakers might be able to more successfully mitigate problematic alcohol use and promote healthier life choices.

## Data Availability

The datasets generated or analyzed during this study are not publicly available due to copyright restrictions, but are available from the corresponding author on reasonable request.

## Abbreviations

CRAFFT-d: German version of Car-, Relax-, Alone-, Forget-, Friends-, Trouble- questionnaire to assess problematic alcohol use
HLS-EU-Q16: European Health Literacy Survey instrument
